# Supplementary material for: Prevalence and predictors of use of long-term and short-acting reversible contraceptives among women of reproductive age in Wakiso and Hoima districts, Uganda: A cross-sectional study
Source: PLOS Glob Public Health. 2023 Dec 11;3(12):e0002688. doi: 10.1371/journal.pgph.0002688 (PMC10712849; doi:10.1371/journal.pgph.0002688)
Supplement: S1 Text — (DOCX) [file pgph.0002688.s002.docx]

S1 Text: List of Legends

| baseline_sex | Sex of participant at baseline |
| --- | --- |
| baseline_sexever | Ever had sex at baseline |
| baseline_urbanity | Baseline information on place of residence (urbanicity) |
| baseline_schooling | Participant schooling |
| baseline_age_cat_5 | Baseline age category |
| baseline_age_cat_10 | Baseline age category |
| baseline_age_cat_65_10 | Baseline age category |
| baseline_sexever2 | had sex at baseline coded |
| age_PHS2_agefbirth | Age at first birth second follow up (PHS2-Population Health Surveillance) |
| baseline_ageyrsc | Baseline age in years |
| baseline_marage | Marital status at baseline |
| baseline_birthno | Baseline childbirth number |
| baseline_agesexd | Age at first sex (baseline) |
| PHS2_contraceptivetrad | Contraceptive use – traditional methods |
| PHS2_contraceptiveshort | Contraceptive use-short-acting reversible methods |
| PHS2_contraceptivelong | Contraceptive use-long-term methods |
| PHS2_contraceptiveNO | No contraceptive use |
| PHS2_currentuseMethods | Current contraceptive use methods (not code named) |
| PHS2_currentuse3 | Current contraceptive use methods (codenamed) |
| PHS2_birthnos | Number of children given during second PHS follow-up |
| PHS2_religions | Religious affiliations |
| womens_age PHS2_marcurr2 | Age at current marriage |
| age_firstmarage | Age at first marriage |
| age_firstsex | Age at first sex (follow-up survey) |
| PHS2_fpdecide3 | Who decides on use of family planning methos |
| baseline_religion2 | Religious affiliations |
| region_2 | Regions |
| PHS2_currentusemodern | Current use of modern contraceptives |
| PHS2_cfp_long | Contraceptive use – long-term methods |
| PHS2_fpshortA_RC | Contraceptive use-short-acting reversible methods |
| PHS2_fpTRAdCur | Contraceptive use-long-term methods |
| PHS2_fpNO_All_fp | No contraceptive use |
| PHS2_currenTfp_USE3 | Current contraceptive use methods (not code named) |
| no_of_children | Current contraceptive use methods (codenamed) |
| PHS2_cfp_LARC | Long-term contraceptive use (follow-up survey 2) |
| PHS2_fpshortA_RCAA | Short-term contraceptive use (follow-up survey 2) |
| PHS2_fpTRAdCurAA | Short-term contraceptive use (follow-up survey 2) |
| PHS2_currenTfp_USE3AA | Current contraceptive use (after elimination of non-response) |
| Level_Schooling | Highest level of schooling |
| Age_cat |  |

sssss
