# Supplementary material for: Prevalence and predictors of use of long-term and short-acting reversible contraceptives among women of reproductive age in Wakiso and Hoima districts, Uganda: A cross-sectional study
Source: PLOS Glob Public Health. 2023 Dec 11;3(12):e0002688. doi: 10.1371/journal.pgph.0002688 (PMC10712849; doi:10.1371/journal.pgph.0002688)
Supplement: Attachment — Submitted filename: Response to reviewer..docx.docx [file pgph.0002688.s003.docx]

**Authors’ response to the editor and reviewer**

**Title:** **Prevalence and predictors of use of long-term and short-acting reversible contraceptives among women of reproductive age in Wakiso and Hoima districts, Uganda: a cross-sectional study**

Authors: Malachi Ochieng Arunda^1^, Babirye Mary Estellah^1^, Carl Fredrik Sjöland^1^, Emmanuel Kyasanku^2^, Stephen Mugamba^2^, Vitalis Ofumbi Olwa^2^, Robert Bulamba^2,4^, Phillip Kato^2^, James Nkale^2^, Fred Nalugoda^2^, Grace Nalwoga Kigozi^2^, Gertrude Nakigozi^2^, Godfrey Kigozi^2^, Joseph Kagaayi^2,4^, Deusdedit Kiwanuka^2^, Stephen Watya^2^, Anna Mia Ekström^1,3^, Elin C. Larsson^1,5^.

Date: October 03 2023

Authors responses:

See over

**Dear editor**

**Re: Prevalence and predictors of use of long-term and short-acting reversible contraceptives among women of reproductive age in Wakiso and Hoima districts, Uganda: a cross-sectional study**

**(PGPH-D-22-01907)**

We have examined the queries by the reviewer and attached a point-by-point responses to the comments. Senior co-authors from both Sweden and Uganda have also done thorough review of this manuscript and we have made worthwhile changes.

We have included the unmarked revised copy labeled `Manuscript´ and a marked-up file labeled `Revised Manuscript with Tracked Changes´ as required.

We look forward to hearing from you.

Kind regards,

Malachi Ochieng Arunda

Corresponding author

On behalf of all co-authors

**Authors´ responses**

**Editor**

**Data availability statement.**

As required, the data availability statement has been amended in the online submission form to *“All the data underlying the findings described in the manuscript has been made available”.*

The dataset has been added as a supplementary information.

**Patients and public involvement**

The public was only involved as research participants. They were not directly involved in the design, or recruitment, conduct, reporting, or dissemination plans of this study.

**Reviewer**

**Comment #1.** Overall, this is a well-written manuscript on a highly relevant and useful topic. Please see my comments and questions in the attached version of your manuscript. Many of them are minor suggestions.

**Response:** Thank you for the minor grammatical changes proposed. We have now made correction in the whole manuscript based on those suggestions. Additionally, we have also conducted thorough multiple co-author reviews and made additional changes to improve the manuscript. The changes can be seen in the “Revised Manuscript with Tracked Changes”.

**Comment#2.** Please review my comments on your conclusions section. It is unclear how you reached the conclusion that expanded family planning clinics and mobile clinics are needed from your data analysis. What conclusions can you reach strictly from this analysis?

**Response:** The have made changes in the conclusion and included on comments based on our study findings. It now reads *“About half of the women in the study used modern contraceptives and the use of SARC was almost twice that of long-term methods. Increased access to contraception education for all women of reproductive age could significantly improve the use of long-term methods which offer more reliable protection against unintended pregnancies. The findings shed light on the need to strengthen both general and sexuality education to girls and women and to tailor contraception access for all in need, for mobile semi-urban as well as rural women. Well-informed strategies that engage young men and male partners in informed decision-making for contraceptive use could enhance progress*.*”*

End
